# Coronary Interventions in Pediatric Congenital Heart Disease

**DOI:** 10.1007/s00246-021-02784-x

**Published:** 2021-12-13

**Authors:** Anastasia Schleiger, Peter Kramer, Stephan Dreysse, Stephan Schubert, Björn Peters, Joachim Photiadis, Felix Berger, Johannes Nordmeyer

**Affiliations:** 1 Department of Congenital Heart Disease/Pediatric Cardiology, German Heart Centre Berlin, Augustenburger Platz 1, 13353 Berlin, Germany; 2 Department of Cardiology, German Heart Centre Berlin, Berlin, Germany; 3 Herz- und Diabeteszentrum NRW - Clinic for Pediatric Cardiology and Congenital Heart Defects, Bad Oyenhausen, Germany; 4 Department of Congenital Heart Surgery/Pediatric Heart Surgery, German Heart Centre Berlin, Berlin, Germany; 5 DZHK (German Center for Cardiovascular Research), Partner Site Berlin, Berlin, Germany; 6 Division of Cardiology, Department of Pediatrics, Charité-Universitätsmedizin Berlin, Berlin, Germany

**Keywords:** Percutaneous coronary intervention, Coronary artery stenosis, Coronary fistula, Cardiac allograft vasculopathy, Pediatric congenital heart disease

## Abstract

Coronary artery lesions represent rare conditions in pediatric congenital heart disease and mainly include coronary artery stenoses (CAS) or coronary artery fistulae (CAF). Due to the small vessel size, pediatric percutaneous coronary interventions (PCI) are demanding and studies concerning long-term results are missing. In this retrospective study, we analyzed indications, procedural details, and post-procedural outcomes in pediatric patients who underwent PCI in our institution. For CAS treatment, procedural success was defined as efficient coronary revascularization with a significant improvement of coronary perfusion. CAF treatment was considered successful, when no residual shunt was detectable. From 1995 to 2020, 32 pediatric patients aged ≤ 18 years received interventional treatment for CAS (*n* = 24/32) or CAF (*n* = 8/32). Reasons for CAS were post-surgical (*n* = 15/24) or post-transplant (*n* = 9/24). Interventional treatment strategies included coronary angioplasty (20/43), stent placement (10/43), and a combination of both (13/43). In-hospital mortality occurred in 6/24 patients and late mortality in 5/24 patients leading to an overall 5-year survival of 62.5%. Early mortality mainly occurred due to post-ischemic myocardial failure. CAF occlusion was performed using coil embolization (*n* = 3), placement of vascular plugs (*n* = 3), a combination of both (*n* = 1), or a combination of coil embolization and a covered stent (*n* = 1). Treatment of coronary fistulae was successful in all patients with excellent post-procedural results and no follow-up death. PCI in pediatric patients with congenital heart disease can be performed safely and effectively. However, the overall 5-year survival probability of patients with CAS is reduced due to severe ischemic myocardial damage.

**Supplementary Information:**

The online version contains supplementary material available at 10.1007/s00246-021-02784-x.

## Introduction

Coronary artery lesions represent a rare condition in pediatric congenital heart disease and mostly include coronary artery stenoses (CAS) and coronary artery fistulae (CAF) [1, 2]. CAS mainly occur after surgical coronary artery translocation or due to cardiac allograft vasculopathy (CAV) after pediatric cardiac transplantation [3, 4]. Coronary artery translocation represents the most challenging surgical part of the arterial switch operation (ASO) for anatomic correction of transposition of the great arteries [5]. Despite excellent short- and long-term results, the incidence of CAS after ASO is reported to be 3–11% with a significant impact on morbidity and mortality [1, 3, 6]. CAV is a serious complication after pediatric cardiac transplantation accounting for more than 25% of late deaths and being the major indication for retransplantation [7]. Despite extensive experience in adult patients, percutaneous coronary interventions (PCI) are rare interventional procedures in the pediatric population and studies describing short- and long-term results are missing.

CAF are defined as abnormal connections between coronary arteries and a cardiac chamber. The true incidence of CAF in the pediatric population is unknown since most CAF do not cause symptoms and thus are clinically undetectable [2]. However, when diagnosed occlusion is recommended in large CAF to avoid long-term complications, such as myocardial ischemia, congestive heart failure, endocarditis, or rupture of a fistulous aneurysm [2, 8]. Various interventional techniques and occlusion devices have been described for transcatheter closure of CAF with excellent results [9, 10]. However, due to the small vessel size, coronary interventions in children are demanding and require considerable theoretical and practical expertise.

The aim of this study was to analyze the feasibility, interventional techniques, and procedural success of PCI performed to treat CAS and CAF in our institution.

## Patients and Methods

### Study Design and Study Endpoints

We retrospectively identified 32 pediatric patients aged ≤ 18 years who received interventional treatment for coronary lesions in our institution between January 1995 and August 2020. The study was approved by the institutional review board and ethics committee (decision number: EA2/009/21). Informed consent was not mandatory due to the retrospective character of this study. In-hospital mortality was defined as death before hospital discharge or within 30 days after an interventional treatment. Accordingly, late mortality was classified as occurring after more than 30 days after an interventional procedure. Interventional treatment of CAS was considered successful when efficient coronary revascularization with a significant improvement of coronary perfusion and lumen gain in the treated vessel was achieved. For the treatment of CAF, procedural success was defined as absent or mild residual shunt.

### Interventional Treatment of CAS

Indications for selective coronary angiography were based on clinical as well as electrocardio- or echocardiographic signs of impaired myocardial perfusion (e.g., reduced cardiopulmonary capacity, ST-segment anomalies, impaired ventricular contractility, or unsuccessful weaning from cardiopulmonary bypass). Cardiac catheterization was performed using the combination of local anesthesia and conscious sedation, and vascular access was obtained via sheath placement in the femoral artery (4- to 5-Fr introducer sheath, Terumo, Tokyo, Japan). A bolus of Heparin (100 units/kg body weight) was applied to avoid catheter-induced thrombus formation. Selective coronary angiography was performed using appropriate diagnostic or guiding catheters (either a modified right Amplatz catheter (Cordis®, Miami, Florida, USA) or a right or left Judkins catheter (JL 3.0–4.0 or JR 3.0–4.0, Cordis®, Miami, Florida, USA), 4–5 Fr corresponding to the sheath size. Prior to PCI 0.1 mg nitroglycerin was applied into the coronary artery to avoid vascular spasm. PCI was performed after selective coronary angiography using a 0.0014-inch coronary wire (Balance MiddleWeight, BMW, Abbott, Chicago, Illinois, USA). Based on the size of the coronary artery either monorail or over-the-wire coronary balloon catheter ranging from 1.5 to 3.5 mm was advanced and balloon angioplasty of the stenosis, primary stent placement, or the combination of both were performed (Supplemental Table 1). After PCI, a final angiography determined procedural success. After PCI all patients received dual antiplatelet therapy with aspirin (3–5 mg/kg body weight) and clopidogrel (0.2 mg/kg body weight) for a period of 12 months, followed by a lifelong monotherapy with aspirin.

### Interventional Treatment of CAF

Indications for selective coronary angiography were clinical signs of congestive heart failure (e.g., failure to thrive, tendency of respiratory infections, impaired cardiopulmonary exercise capacity), echocardiographic evidence of diastolic flow reversal in the aorta, echocardiographic signs of volume overload or an impaired systolic ventricular function, or electrocardiographic signs of myocardial steal or reduced myocardial perfusion. Anatomic features of CAF were determined using selective coronary angiography as described in the section above. In high-flow coronary fistulae a balloon occlusion angiography was performed using a Berman angiographic catheter (Teleflex®, Morrisville, USA). A BMW or Crosswire® NT (Terumo, Tokyo, Japan) were mainly used as a guide wire. Appropriate diagnostic or guiding catheters (either a modified right Amplatz catheter (Cordis®, Miami, Florida, USA) or a right or left Judkins catheter (JL 3.0–4.0 or JR 3.0–4.0, Cordis®, Miami, Florida, USA) were placed at the site of occlusion prior to coil or device implantation. Catheter approach and occlusion devices used for CAF closure varied based on patient and coronary artery size and fistula anatomy, including its origin and drainage, size, side branches and structure (Supplemental Table 2). Antegrade approach was performed via femoral or jugular vein and retrograde approach via femoral artery. To facilitate the antegrade approach, in some patients, an arteriovenous wire loop was formed by advancing a 0.0035-inch guidewire (Radiofocus®. Terumo, Tokyo, Japan) from the aortic root through the fistula into the right atrium and snaring the guidewire from the venous end. Periprocedural antibiotic prophylaxis was performed 30 min prior to device placement using cefazolin (30 mg/kg body weight). After device placement, control angiographies determined procedural success. After treatment, patients received a monotherapy with aspirin (3–5 mg/kg body weight) or a dual antiplatelet therapy or warfarin for a period of 6 to 12 months.

### Statistical Analysis

Data were obtained from medical records of the German Heart Center Berlin. Patient’s characteristics are expressed as median and interquartile range [IQR 25th percentile; 75th percentile]. Survival and freedom from reintervention were assessed using Kaplan–Meier survival analysis. Differences between groups were analyzed using the Log-Rank test. Statistical analysis was performed using SPSS statistical software program (version 23, IBM Corp., NY, USA). A *p-*value < 0.05 was considered statistically significant.

## Results

### Patient Characteristics

From January 1995 to August 2020, 32 pediatric patients received interventional treatment for coronary lesions. Twenty-four patients were diagnosed with CAS and eight patients with CAF. Patient characteristics of both subcohorts are listed in Supplemental Tables 1 and 2. Briefly, median patient age at first coronary intervention was 6.0 years [IQR 2.3; 14.2] and median patient weight was 19.0 kg [IQR 11.5; 48.8]. Median follow-up time was 8.3 years [IQR 2.9; 13.8].

### Coronary Artery Stenosis

**Fig. 1:**
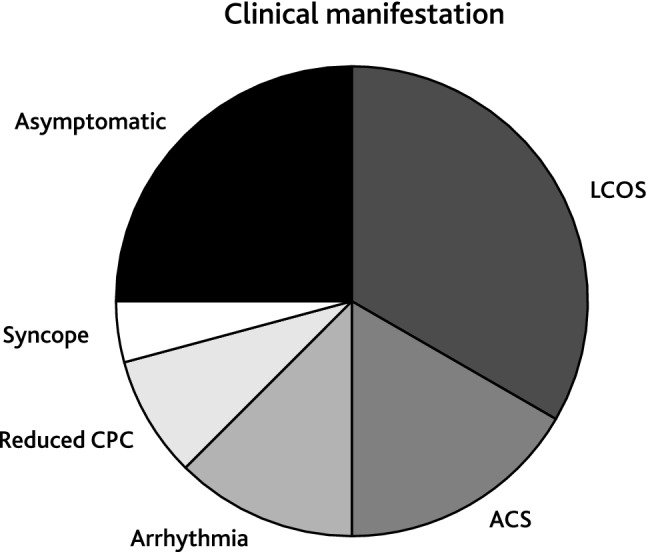
Pie chart depicting clinical presentation/symptoms of coronary artery stenoses (CAS) of the total cohort. *ACS* acute coronary symptom, *CPC* cardiopulmonary capacity, *LCOS* low cardiac output

**Fig. 2:**
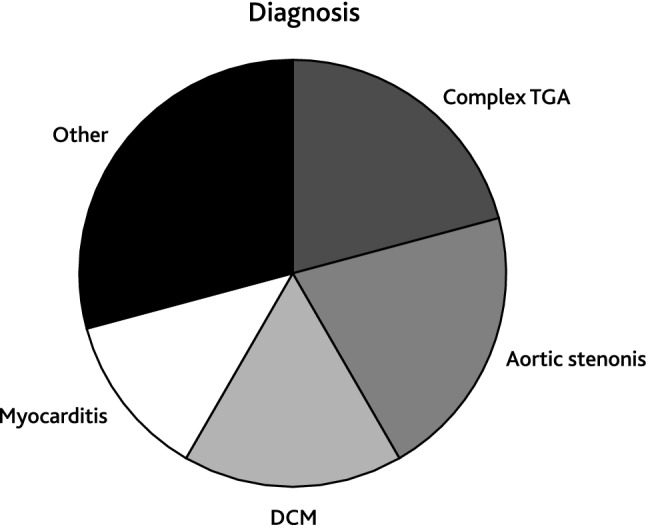
Pie chart depicting the underlying morphologies of patients with coronary artery stenoses (CAS). *DCM* dilated cardiomyopathy, *TGA* transposition of the great arteries

**Fig. 3:**
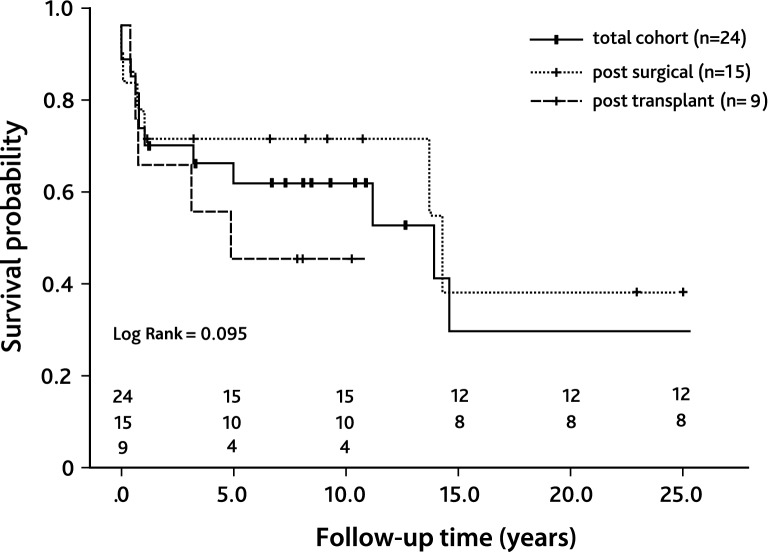
Kaplan–Meier curve depicting freedom from reinterventions after interventional treatment of CAS. The black line represents the total cohort (*n* = 24), the dashed line represents patients with post-transplant CAS (*n* = 9), and the dotted line represents patients with post-surgical CAS (*n* = 15). *CAS* coronary artery stenosis

**Fig. 4:**
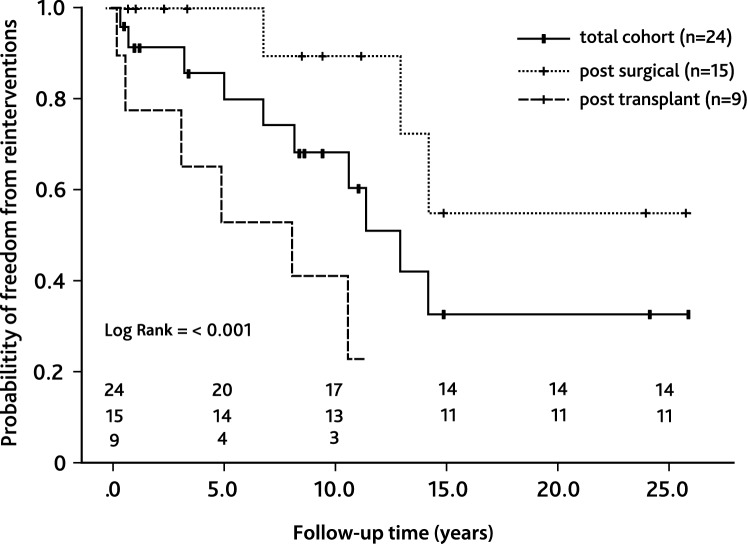
Kaplan–Meier curve depicting overall survival after interventional treatment of CAS. The black line represents the total cohort (*n* = 24), the dashed line represents patients with post-transplant CAS (*n* = 9), and the dotted line represents patients with post-surgical CAS (*n* = 15). *CAS* coronary artery stenosis

Of the entire cohort, 24 patients received 43 interventional procedures to treat CAS. Patient characteristics of this subcohort are listed in Supplemental Table 1. Median patient age at first intervention was 6.7 years [IQR 1.8; 14.8] and median patient weight was 20.7 kg [IQR 9.5; 46.1]. The smallest patient who received PCI weighted 3.0 kg. Most common underlying cardiac morphologies were complex transposition of the great arteries (*n* = 5), congenital aortic valve stenosis (*n* = 5), dilated cardiomyopathy (*n* = 4), and myocarditis (*n* = 3, Fig. 1). Causes of CAS were post-surgical (*n* = 15) or post-transplant (*n* = 9). The majority of patients diagnosed with CAS presented with low cardiac output (*n* = 8), 7 of these patients required mechanic circulatory support. Other symptoms were acute coronary syndrome (*n* = 4), arrhythmia (*n* = 3), reduced cardiopulmonary exercise capacity (*n* = 2), or syncope (*n* = 1, Fig. 2). Six patients were asymptomatic and CAS was diagnosed during routinely performed cardiac catheterization (Fig. 2). Treatment of CAS included 43 PCIs consisting of 20 balloon angioplasties, 10 stent placements (drug-eluting stent: *n* = 14; bioresorbable stent: *n* = 1, Supplement Table 1), and 13 combinations of both. One vessel was addressed in 18 patients (LAD: *n* = 11; RCA: *n* = 6; CX: *n* = 1) and 2 vessels in 6 patients (LAD + CX: *n* = 4; CX + RCA: *n* = 1, RCA + LAD: *n* = 1; Supplemental Table 1). Procedural success was achieved in 23 of 24 patients. Median follow-up time after CAS treatment was 7.4 years [IQR 0.8; 10.1]. Ten patients required one or more reinterventions due to a significant re-stenosis after a median time of 10.1 months [IQR 0.5; 10.1] after initial treatment. Freedom of reintervention was 62.6% after 5 years (Fig. 3). In patients with post-surgical CAS the reintervention rate was significantly lower compared to patients with post-transplant CAS (post-surgical: 3/15; post-transplant: 7/9, Log-Rank test: *p* < 0.001, Fig. 3). In-hospital mortality occurred in 6 patients and late mortality in 5 patients resulting in an overall 5-year survival probability of 62.5% after interventional treatment of CAS (Fig. 4). Mortality rate did not significantly differ between patients with post-surgical and post-transplant CAS (post-surgical: 6/15; post-transplant: 6/9, Log-Rank test: *p* = 0.095, Fig. 3). In patients with post-surgical CAS, major causes of death were ischemic myocardial damage, which was irreversible even after a successful interventional revascularization. Whereas, in post-transplant patients death mainly occurred due to graft-loss based on progression of CAV. Five patients suffered from cardiac arrest and required cardiopulmonary resuscitation and mechanical circulatory support prior to primary interventional treatment. In two patients cardiopulmonary resuscitation and placement of mechanical circulatory support occurred as a complication of interventional treatment. Both patients developed a hemodynamically relevant 3rd degree atrioventricular block during selective intubation and wire placement into the left coronary artery leading to low cardiac output and cardiac arrest.

### Coronary Artery Fistula

CAF was diagnosed in 8 of 32 patients who received interventional treatment for coronary lesions. Patient characteristics of this subcohort are listed in Supplemental Table 2. Median patient age was 3.9 years [IQR 1.8; 12.6] and median patient weight was 16.2 kg [IQR 12.3; 53.2]. Associated cardiac morphologies were single coronary anatomy (*n* = 2), tetralogy of Fallot (*n* = 1) and pulmonary atresia with intact ventricular septum (*n* = 1). Clinical symptoms were found in 4 of 8 patients. One patient had experienced ventricular tachycardia, 2 patients displayed recurrent respiratory infections, and 1 patient had failure to thrive and impaired cardiopulmonary exercise capacity. Four of eight patients were asymptomatic but showed echocardiographic flow reversal in the aorta, signs of volume overload, or impaired ventricular contractility performance. The fistulae originated from the LCA in 4 patients and from the RCA in 3 patients. One patient (Supplemental Table 2, No. 31) was diagnosed with 2 coronary fistulae originating from both LCA and RCA. CAF drained to the right atrium in 3 patients and to the right ventricle in 5 patients. One patient (Supplemental Table 2, No. 29) had undergone previous surgical treatment of CAF but displayed significant residual shunt. Transcatheter fistulae occlusion was performed via antegrade catheter approach in 2 patients, which was facilitated using arteriovenous wire loop technique in additional 2 patients, and retrograde catheter approach in 3 patients. One patient (Supplemental Table 2, No. 25) received distal fistula occlusion via antegrade and proximal fistula occlusion via retrograde catheter approach. Occlusion of CAF was performed using coil embolization (*n* = 3), placement of vascular plugs (*n* = 3), a combination of both (*n* = 1), or a combination of coil embolization and a covered stent (*n* = 1, Supplemental Table 2). CAF treatment was successful in all patients with no residual shunt in 7 patients and mild residual shunt in 1 patient, which was unverifiable during follow-up cardiac catheterization 6 months after initial treatment. The median follow-up time after transcatheter closure of CAF was 12.3 years [IQR 3.9; 16.3], and no death or reintervention occurred until follow-up end.

## Discussion

In this study we retrospectively analyzed the indications, techniques, and outcome of pediatric coronary interventions performed in our institution, including the interventional treatment of CAS and CAF.

### Coronary Artery Stenoses

Post-surgical and post-transplant CAS represented the major indications for coronary interventions in our patient cohort. Post-surgical CAS mainly occurred after coronary artery translocation during ASO or surgical correction of congenital aortic valve stenosis [1, 3, 11]. The reasons for post-surgical CAS vary from coronary anomalies, coronary ostium distortion, coronary artery compression, and thrombus or abnormal scar tissue formation [12]. In this subcohort, a high number of patients presented with low cardiac output requiring cardiopulmonary resuscitation and mechanic circulatory support. Our results revealed a high in-hospital mortality rate in this particular patient cohort despite successful interventional revascularization in the majority of patients. This observation might be caused by the severe ischemic myocardial damage prior to PCI, which—in some patients—is severe and therefore irreversible even when sufficient revascularization is achieved. Since unfortunately no general recommendations exist concerning the treatment of CAS in pediatric patients, our institutional approach included PCI with balloon angioplasty and/or stent placement depending on the individual findings of type, extent, and location of CAS. In patients with immediate post-surgical CAS balloon diameters slightly smaller than the stenosis were preferred and ruptures of surgical sutures were successfully avoided. Even if some study groups suggest that considering the mechanical features of scar tissue formation an overextension might lead to a more beneficial lumen gain after a longer post-operative period [12] and an overextension was strictly avoided at our institution. Especially in small pediatric patients balloon angioplasty was the most favored revascularization technique, whereas stent implantation was preserved for patients with insufficient revascularization results to avoid in-stent re-stenosis. However, primary coronary stent placement with excellent acute implant success has been described [13]. To date, the optimal interventional strategy in treatment of CAS in pediatric patients still remains controversial. However, balloon angioplasty alone leads to adequate growth of coronary arteries years after interventions and no data exist concerning the requirement and possibilities of stent re-dilatation to keep up with somatic growth in coronary arteries [12].

CAV is the leading cause of late graft failure, mortality, and retransplantation in pediatric heart transplant recipients with an incidence of 35% 10 years after cardiac transplantation [7, 14]. Coronary revascularization strategies are a major component of the multimodal therapeutic treatment of CAV [14]. While in adult heart transplant recipients, stent implantation is the preferred treatment option to improve short-term outcome no data concerning an effective revascularization strategy exist for the pediatric population [14, 15]. Jeewa et al. [16] reported an overall procedural success rate of 73% after PCI with stent implantation; however, graft failure occurred in 39% of patients within 1 year after interventional treatment. In our cohort, 7 of 9 cardiac transplantation recipients receiving interventional treatment of CAV required frequent reinterventions. Graft failure occurred in 6 of 9 patients (retransplantation: *n* = 1, death: *n* = 5) after a median time of 1.97 years [IQR 5.39] after the first reintervention. These results might indicate that coronary interventions, e.g., PTCA and stent placement might delay but not prevent graft failure and the timing of retransplantation. However, more studies are needed to investigate interventional treatment options for CAV in children. Evidence is limited by the small number of patients due to the rarity of the disease and interventional addressment of lesions is limited by the unavailability of adequate material tailored to the pediatric population with regard to lengths and diameters.

### Coronary Artery Fistulae

CAF are defined as an abnormal connection between one or more coronary arteries and a cardiac chamber [2]. Whereas the RCA is described to be the most frequent origin of CAF, in our cohort 50% of CAF originated from the LCA and all CAF drained into the right heart chambers. Half of our patients developed clinical symptoms leading to the diagnosis of CAF, whereas in the other half CAF was diagnosed by echocardiography performed to evaluate a heart murmur. These findings underline the important role of echocardiography in the diagnosis of CAF. Elective transcatheter occlusion of large CAF has been recommended to avoid complications, such as congestive heart failure, growth impairment, rupture of a fistula aneurysm, or endocarditis [8–10]. Our techniques for CAF occlusion varied basing on fistula anatomy and size and included retrograde arterial and antegrade venous approaches or the arteriovenous wire loop technique. For CAF occlusion, coil embolization or device placement was utilized, and some patients required a combination of both. Our results have shown that closure of CAF can be performed with calculable risk, no peri-procedural complications, no mortality and excellent results. Due to the feasibility, safety, and efficiency of transcatheter closure of CAF we prefer an interventional over a surgical approach, which has been reported to be the “gold standard” by other institutions [17].

## Conclusion

Transcatheter closure of CAF in children can be performed safely and with excellent procedural success and long-term outcome. In patients with CAS interventional revascularization is feasible and leads to excellent post-procedural results. However, early- and mid-term survival rates are lower due to the urgency and severity of the underlying disease, its complications, and limited causal treatment options.

### Limitations

There are several limitations to this study. This is a retrospective, single-center study with a small patient cohort. Further studies, preferably with a larger patient cohort and in a multi-center setting, are needed to evaluate interventional treatment options and procedural success in pediatric patients with coronary artery lesions. Angiographic imaging was not available for every patient of our cohort, therefore procedural outcomes were assessed by analyzing medical reports. The absence of angiographic imaging impeded a more precise definition of procedural success after CAS treatment in our manuscript. Additionally, due to the unique anatomy and rarity of coronary artery lesions in pediatric patients no universal recommendations exist concerning interventional techniques and procedural strategies leading to an individual institutional approach, which complicates the establishment of guidelines.

## Supplementary Information

Below is the link to the electronic supplementary material.

Supplementary file1 (DOCX 23 KB)

## Abbreviations

ASO: Arterial switch operation
CAF: Coronary artery fistula
CAS: Coronary artery stenosis
CAV: Cardiac allograft vasculopathy
ECG: Electrocardiogram
Fr: French
LAD: Left anterior descending artery
LCA: Left coronary artery
PCI: Percutaneous coronary intervention
RCA: Right coronary artery
CX: Circumflex artery

## References

[1] Bonhoeffer P, Bonnet D, Piechaud JF, Stumper O, Aggoun Y, Villain E (1997). Coronary artery obstruction after the arterial switch operation for transposition of the great arteries in newborns. J Am Coll Cardiol.

[2] Loukas M, Germain AS, Gabriel A, John A, Tubbs RS, Spicer D (2015). Coronary fistula: a review. Cardiovasc Pathol.

[3] Tsuda T, Bhat AM, Robinson BW, Baffa J, Radtke W (2015). Coronary artery problems late after arterial switch operation for transposition of the great arteries. Circ J.

[4] Pahl E, Naftel DC, Kuhn MA, Shaddy RE, Morrow WR, Canter CE (2005). The impact and outcome of transplant coronary artery disease in a pediatric population: a 9-year multi-institutional study. J Heart Lung Transplant.

[5] Castaneda AR, Norwood WI, Jonas RA, Colan SD, Sanders SP, Lang P (1984). Transposition of the great arteries with intact ventricular septum: anatomical repair in the neonates. Ann Thorac Surg.

[6] Losay J, Touchot A, Serraf A, Litvinova A, Lambert V, Piot JD (2001). Late outcome after arterial switch operation for transposition of the great arteries. Circulation.

[7] Nandi D, Chin C, Schumacher KR, Fentin M, Singh RK, Kimberly Y (2020). Surveillance for cardiac allograft vasculopathy: practice variations among 50 pediatric heart transplant centers. J Heart Lung Transplant.

[8] Armsby LR, Keane JF, Sherwood MC, Forbess JM, Perry SB, Lock JE (2002). Management of coronary artery fistulae. patient selection and transcatheter closure. J Am Coll Cardiol.

[9] Quereshi SA, Tynan M (2001). Catheter closure of coronary artery fistulas. J Interv Cardiol.

[10] Abdelmoneim SS, Mookadam F, Moustafa S, Zehr KJ, Mookadam M, Maaloof JF (2007). Coronary artery fistula: Single-center experience spanning 17 years. J Interv Cardiol.

[11] d’Udekemp Y, Siddiqui J, Seaman CS, Konstantinov IE, Galati JC, Cheung MH (2013). Long-term results of a strategy of aortic valve repair in the pediatric population. J Thorac Cardiovasc Surg.

[12] Kampmann C, Kuroczynski W, Trübel H, Knuf M, Schneider M, Heinemann MK (2005). Late results after PTCA for coronary stenosis after the arterial switch procedure for transposition of the great arteries. Ann Thorac Surg.

[13] Bratincsak A, Salkini A, El-Said HG, Moore JW (2012). Percutaneous stent implantation into coronary arteries in infants. Catheter Cardiovasc Interv.

[14] Kindel SJ, Pahl E (2012). Current therapies for cardiac allograft vasculopathy in children. Congenit Heart Dis.

[15] Bader FM, Kfoury AG, Gilbert EM, Barry WH, Humayun N, Hagan ME (2006). Percutaneous coronary interventions with stents in cardiac transplant recipients. J Heart Lung Transplant.

[16] Jeewa A, Chin C, Pahl E, Atz AM, Carboni MP, Pruitt E (2015). Outcomes after percutaneous coronary artery revascularization procedures for cardiac allograft vasculopathy in pediatric heart transplant recipients: a multi-institutional study. J Heart Lung Transplant.

[17] Said SM, Burkhart HM, Schaff HV, Connolly HM, Phillips SD, Suri RM (2013). Late outcome of repair of congenital coronary artery fistulas—a word of caution. J Thorac Cardiovasc Surg.

