# Supplementary material for: Coronary Interventions in Pediatric Congenital Heart Disease
Source: Pediatr Cardiol. 2021 Dec 13;43(4):769–75. doi: 10.1007/s00246-021-02784-x (PMC9005385; doi:10.1007/s00246-021-02784-x)
Supplement: Supplementary file 1 — Supplementary file1 (DOCX 23 KB) [file 246_2021_2784_MOESM1_ESM.docx]

**Table 1. Patient characteristics and interventional treatment strategies* of patients diagnosed with coronary stenosis**

| **Pat. No.** | **Age**  **(years)** | **Weight**  **(kg)** | **Morphology** | **Stenosis morphology** | **Symptoms** | **No. of**  **Reint.** | **Coronary**  **artery** | **PTCA balloon** | **Stent placed** |
| --- | --- | --- | --- | --- | --- | --- | --- | --- | --- |
| 1 | 0.22 | 8.8 | TGA | Post-surgical | LCOS | 1 | LAD | N.a.  2.0 mm |  |
| 2 | 0.05 | 3.0 | TAC | Post-surgical | LCOS | 0 | LCA | Voyager® OTW 1.5 / 15.0 mm |  |
| 3 | 0.8 | 5.18 | TGA | Post-surgical | LCOS | 0 | LAD | Tazuna®  2.0 / 10.0 mm | Xience Alpine®  2.25 / 8.0 mm |
| 4 | 1.52 | 9.0 | DCM | Post-transplant | asymptomatic | 1 | RCA | Sprinter®  2.0 / 12 mm |  |
| 5 | 1.34 | 7.3 | DCM | Post-transplant | asymptomatic | 4 | RCA | Tazuna®  1.25 / 10.0 mm  SeQuent® Please  2.0 / 17 mm  2.0 / 15 mm |  |
| 6 | 2.58 | 11.0 | AVSD | Post-surgical | ACS | 0 | LAD | N.a.  1.5 mm |  |
| 7 | 3.79 | 13.3 | TA | Post-surgical | ACS | 0 | RCA | Pantera® Pro  3.0 mm |  |
| 8 | 0.67 | 5.3 | IAA | Post-surgical | AV block | 0 | RCX | CrossSail®  2.0 / 20 mm  2.0 / 10 mm |  |
| 9 | 3.65 | 16.7 | TGA | Post-surgical | VF | 0 | LAD | Tazuna®  2.0 / 20.0 mm | Xience Pro®  2.25 / 12.0 mm |
| 10 | 4.31 | 15.7 | myocarditis | Post-transplant | asymptomatic | 3 | LAD  RCA | Sprinter®  2.0 / 30 mm  SeQuent® Please  2.0 / 20 mm  2.0 / 30 mm |  |
| 11 | 6.61 | 26.0 | CAS | Post-surgical | ACS | 0 | LCA | N.a.  1.5 mm  2.0 mm |  |
| 12 | 6.84 | 21.3 | CAS | Post-surgical | LCOS | 0 | RCA |  | Xience Alpine®  3.0 / 8.0 mm |
| 13 | 8.77 | 20.0 | CAS | Post-transplant | LCOS | 0 | LAD  RCX | Sprinter®  2.0 / 20 mm  Sprinter®  2.0 / 20 mm  Tazuna®  1.5 / 10.0 mm | Promus^TM^ Element^TM^  2.5 / 24.0 mm |
| 14 | 9.03 | 26.6 | Myocarditis | Post-transplant | asymptomatic | 1 | RCX  RCA | Tazuna®  1.5 / 10.0 mm  Sprinter®  2.0 / 20 mm  SeQuent® Please  2.5 / 30 mm  SeQuent® Please  2.5 / 26 mm  SeQuent® Please  2.5 / 30 |  |
| 15 | 11.74 | 45.0 | TGA | Post-transplant | syncope | 1 | LAD  RCX | Maverick^TM^  2.0 / 20.0 mm  Maverick^TM^ OTW  2.0 / 20.0 mm | Taxus®  3.0 / 12.0 mm  Multi-link MiniVision®  2.0 / 12.0 mm |
| 16 | 13.91 | 51.0 | TGA | Post-surgical | Reduced CPEC | 1 | LAD | Trek®  2.5 / 12.0 mm | Absorb^TM^  3.5 / 12.0 mm |
| 17 | 14.19 | 44.0 | TOF | Post-surgical | Reduced  CPEC | 0 | LAD | Viva^TM^  1.5 / 20.0 mm | Multi-Link Pixel^TM^  2.5 / 13.0 mm |
| 18 | 15.04 | 39.0 | myocarditis | Post-transplant | ACS | 0 | LAD  RCX |  | Multi-Link®  3.5 / N.a.  Multi-Link  3.0 / N.a  Multi-Link  3.0 / N.a |
| 19 | 15.98 | 69.0 | DCM | Post-transplant | asymptomatic | 5 | RCA | Voyager^TM^  2.0 / 20.0 mm |  |
| 20 | 16.88 | 46.5 | PAIVS | Post-surgical | LCOS | 0 | RCA |  | Promus Element^TM^  3.5 / 12.0 mm |
| 21 | 17.46 | 56.0 | ALCAPA | Post-surgical | LCOS | 1 | LAD  RCX | Maverick^TM^ OTW  2.0 / 20.0 mm  Maverick^TM^ OTW  5.0 / 20.0 mm  Maverick^TM^  2.5 / 30.0 mm  Viva^TM^  1.5 / 20.0 mm  OpenSail^®^  1.5 / 20.0 mm | Taxus®  4.5 / 28.0 mm |
| 22 | 17.28 | 53.0 | DCM | Post-transplant | asymptomatic | 1 | LAD |  | Promus®  3.0 / 12.0 mm |
| 23 | 18.95 | 62.0 | CAS | Post-surgical | Arrhythmia | 0 | LAD | Grip^TM^  3.5 / 12.0 mm | Promus Element^TM^  4.0 / 8.0 mm |
| 24 | 5.17 | 18.0 | CAS | Post-transplant | LCOS | 0 | LAD | Sprinter®  2.0 / 12 mm |  |

*data represent information concerning the first coronary intervention and do not contain information concerning reinterventions.

ACS = acute coronary syndrome; ALCAPA = anomalous left coronary artery from the pulmonary artery; AV = atrioventricular; AVSD = atrioventricular septum defect; CAS = congenital aortic stenosis; CPE = cardiopulmonary exercise capacity; DCM = dilated cardiomyopathy; IAA = interrupted aortic arch; LCOS = low cardiac output syndrome; LAD = left anterior descending branch; N.a. = not available; No. = number; Pa. = patient; PAIVS = pulmonary atresia with intact ventricular septum; RCA = right coronary artery; RCX ramus circumflexus; reint. = reintervention; TA = tricuspid atresia; TAC = truncus arteriosus communis, TGA = transposition of the great arteries; TOF = Tetralogy of Fallot; VF = ventricular fibrillation

**Table 2. Patient characteristics and interventional treatment strategies of patients diagnosed with coronary fistula**

| **Pat. No.** | **Age**  **(years)** | **Weight**  **(kg)** | **Associated**  **cardiac**  **morphologies** | **Symptoms** | **Fistula origin** | **Fistula drainage** | **Catheter approach** | **Occlusion device** |
| --- | --- | --- | --- | --- | --- | --- | --- | --- |
| 25 | 0.85 | 8.8 | none | Asymptomatic | RCA | RA | Distal occlusion: antegrade  Proximal occlusion: retrograde | 1. Amplatzer^TM^ Membranous VSD Occluder (6 mm) 2. Cook^©^ Coil (8/5 mm) |
| 26 | 1.52 | 11.7 | none | Tendency to respiratory infections | LCA | RVOT | Arteriovenous wire loop, antegrade device deployment | Amplatzer^TM^ Vascular Plug IV (5 mm) |
| 27 | 2.6 | 16.2 | Monocoronary artery | Asymptomatic | LCA | RVOT | antegrade | Cook^©^ Coil (5/4 mm) |
| 28 | 3.73 | 16.2 | none | Tendency to respiratory infections | RCA | RA | Arteriovenous wire loop, antegrade device deployment | Amplatzer^TM^ Vascular Plug II (12 mm) |
| 29 | 3.97 | 14.0 | Monocoronary artery | Failure to thrive  Impaired exercise capacity | RCX | RVOT | retrograde | 1. 1 Concerto^TM^ Coil (18 mm/ 40 cm) 2. 3 Concerto^TM^ Coils (14 mm/ 30 cm) |
| 30 | 6.81 | 28.3 | none | asymptomatic | RCA | RA | antegrade | Amplatzer^TM^ Vascular Plug II (6 mm) |
| 31 | 18.43 | 66.0 | PA/IVS | Ventricular tachykardia | RCA | RV | retrograde | PK Papyrus Covered Stent^©^ (5/20 mm) |
|  |  |  |  |  | LCA | RV |  | 1. 2 Concerto^TM^ Coil (18 mm/ 40 cm) 2. 2 Concerto^TM^ Coils (16 mm/ 40 cm) |
| 8 | 14.53 | 63.0 | TOF | asymptomatic | LCA | RVOT | retrograde | 1. Coil (2/10 mm) 2. Coil (2/20 mm) |

LCA = left coronary artery; LV = left ventricle; No. = number; Pa. = patient; PAIVS = pulmonary atresia with intact ventricular septum; RA = right atrium, RCA = right coronary artery; RCX = Ramus cirmumflexus; RV = right ventricle; RVOT = right ventricular outflow tract
